# Individual Differences in Intertemporal Choice

**DOI:** 10.3389/fpsyg.2021.643670

**Published:** 2021-04-16

**Authors:** Kristof Keidel, Qëndresa Rramani, Bernd Weber, Carsten Murawski, Ulrich Ettinger

**Affiliations:** ^1^Department of Psychology, University of Bonn, Bonn, Germany; ^2^Department of Finance, The University of Melbourne, Melbourne, VIC, Australia; ^3^Center for Economics and Neuroscience, University of Bonn, Bonn, Germany; ^4^Institute of Experimental Epileptology and Cognition Research, University of Bonn, Bonn, Germany

**Keywords:** decision making, personality, cognition, neuroimaging, molecular genetics

## Abstract

Intertemporal choice involves deciding between smaller, sooner and larger, later rewards. People tend to prefer smaller rewards that are available earlier to larger rewards available later, a phenomenon referred to as temporal or delay discounting. Despite its ubiquity in human and non-human animals, temporal discounting is subject to considerable individual differences. Here, we provide a critical narrative review of this literature and make suggestions for future work. We conclude that temporal discounting is associated with key socio-economic and health-related variables. Regarding personality, large-scale studies have found steeper temporal discounting to be associated with higher levels of self-reported impulsivity and extraversion; however, effect sizes are small. Temporal discounting correlates negatively with future-oriented cognitive styles and inhibitory control, again with small effect sizes. There are consistent associations between steeper temporal discounting and lower intelligence, with effect sizes exceeding those of personality or cognitive variables, although socio-demographic moderator variables may play a role. Neuroimaging evidence of brain structural and functional correlates is not yet consistent, neither with regard to areas nor directions of effects. Finally, following early candidate gene studies, recent Genome Wide Association Study (GWAS) approaches have revealed the molecular genetic architecture of temporal discounting to be more complex than initially thought. Overall, the study of individual differences in temporal discounting is a maturing field that has produced some replicable findings. Effect sizes are small-to-medium, necessitating future hypothesis-driven work that prioritizes large samples with adequate power calculations. More research is also needed regarding the neural origins of individual differences in temporal discounting as well as the mediating neural mechanisms of associations of temporal discounting with personality and cognitive variables.

## Introduction

People frequently have to decide between rewards of different magnitudes that become available at different times in the future. Examples include whether to spend money on an event that is immediately rewarding, such as a night out, or to save it toward a future activity with potentially greater subjective value, such as an overseas holiday. The problem of choosing between smaller, sooner and larger, later rewards is referred to as *intertemporal choice* (Frederick et al., 2002). People generally prefer smaller rewards that are available earlier over larger rewards available later, thereby devaluing future rewards. This phenomenon is known as *temporal* or *delay discounting* (Ainslie, 1975; Frederick et al., 2002). Devaluation of future rewards has far-reaching consequences for wealth and health not only of decision makers themselves, but also society at large (Mischel et al., 1989; Frederick et al., 2002; Golsteyn et al., 2014).

Temporal discounting is highly reproducible in humans and is ubiquitously observed in many other animal species (Berns et al., 2007; Kalenscher and Pennartz, 2008; Stevens, 2011; Frost and McNaughton, 2017). At the same time, temporal discounting has been found to display remarkable variability in magnitude both within and across individuals (Frederick, 2005; Peters and Büchel, 2011).

**Figure 1:**
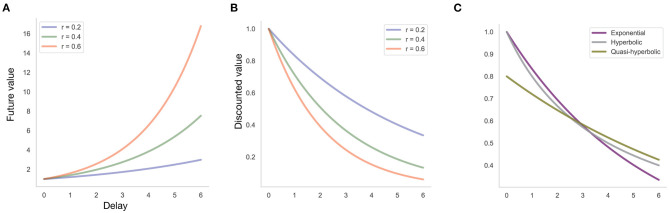
Individual differences in discount functions. **(A)** Future value of $1 received today with exponential discount function at different discount rates *r*, that is, the value of an amount received after a given delay that is equivalent to $1 received today. This is the amount at which a decision-maker with a given discount function is indifferent between receiving this amount at a given delay, and $1 received today. **(B)** Discounted value of $1 received at a given delay, assuming an exponential discount function with discount rate *r*, that is, the value to the decision-maker today of $1 received at a given delay. **(C)** Discounted value of $1 received at a given delay, assuming different discount functions (exponential function with discount rate *r* = 0.2, hyperbolic discount function with discount rate *k* = 0.25, quasi-hyperbolic discount function with parameters β = 0.8, δ = 0.9).

Regarding within-subject variability, the magnitude of temporal discounting, indexed by measures of the discount factor (Figure 1), can be reliably influenced by various experimental manipulations, including framing effects and cognitive strategies aimed at future thinking (Lempert and Phelps, 2016; Rung and Madden, 2018; Scholten et al., 2019). Moreover, developmental effects have been well-described, with the extent of temporal discounting decreasing from childhood into early adulthood (Achterberg et al., 2016).

Importantly, a growing body of evidence suggests that intertemporal choice is also subject to substantial *inter-individual variability* (Harrison et al., 2002; Shamosh and Gray, 2008; Mahalingam et al., 2014). These individual differences are for the most part not random or noise, but have instead been found to display both significant temporal stability and heritability. An important basis of this assumption is the test–retest reliability of intertemporal choice, which has been addressed in a number of studies. Specifically, test–retest correlations of measures of intertemporal choices are in the range of 0.5–0.8 and remain significant for test–retest intervals of up to 1 year (Beck and Triplett, 2009; Kirby, 2009; Jimura et al., 2011; Matusiewicz et al., 2013; Martínez-Loredo et al., 2017). Whilst such reliabilities are broadly comparable to those of other measures of cognitive function (Wöstmann et al., 2013; Buelow and Barnhart, 2018), it is clear that they are not perfect and, thereby, impose an upper limit on correlations that may be observed between intertemporal choice and other measures (Spearman, 1904). However, further evidence for the validity of an individual differences approach to intertemporal choice comes from the behavioral genetics literature. Specifically, twin studies have estimated genetic influences to account for approximately 50% of variance in temporal discounting (Anokhin et al., 2011, 2015; Isen et al., 2014); these data further underscore the stability and trait-like nature of the individual differences in intertemporal choice, justifying investigations of their covariation with other traits.

Individual differences in temporal discounting are also associated with numerous health-related outcomes. For example, higher rates of temporal discounting have been shown to be related to a reduced likelihood to check blood pressure, obtain cholesterol testing, attend dental visits, exercise, receive flu shots, engage in safe sexual behavior, and be medically adherent (Bickel, 2015). Higher rates of discounting have also been demonstrated in relation to psychiatric disorders including substance dependence disorders, attention-deficit/hyperactivity disorder, schizophrenia, major depressive disorder, problem gambling, and obesity (Amlung et al., 2019).

Regarding non-health-related behaviors and outcomes, people who discount to a greater degree spend less time searching for a good job (DellaVigna and Paserman, 2005), experience more shallow wage growth (Munasinghe and Sicherman, 2006), take up welfare programs later (Fang and Silverman, 2006), have lower credit scores (Meier and Sprenger, 2012), borrow more on credit cards (Meier and Sprenger, 2010), are more likely to default on their loans (Meier and Sprenger, 2012), are less likely to wear a seat belt (Bickel, 2015), and are more likely to text while driving (Hayashi et al., 2015).

Therefore, thorough characterization of the determinants of individual differences in temporal discounting is warranted. Such work is important not only to identify factors that covary with temporal discounting, but also to uncover the position of this phenomenon within the architecture of cognition and personality more widely and, importantly, to inform theories of the mechanisms that underlie this phenomenon (for the role of individual differences in theory development, see Cronbach, 1957; Underwood, 1975; Cohen, 1994).

The study of individual differences has a long tradition in psychology. Whilst traditionally focused on constructs such as personality and intelligence, there is also a substantial and growing body of evidence on individual differences in measures from cognitive psychology and cognitive neuroscience. Specifically, it has been argued that studies of (inter-)individual differences in those fields complement experimental studies that typically focus on intra-subject effects (Unsworth, 2019). Exemplified by the seminal work of Miyake et al. (2000), numerous studies have adopted individual differences approaches to examine, amongst others, the structure of executive functions (Friedman and Miyake, 2017), working memory (Engle, 2018), inhibitory control (Stahl et al., 2014), attention (Willems and Martens, 2016), and long-term memory (Unsworth, 2019), thereby integrating often separate approaches from differential and experimental psychology (Cohen, 1994).

**Figure 2:**
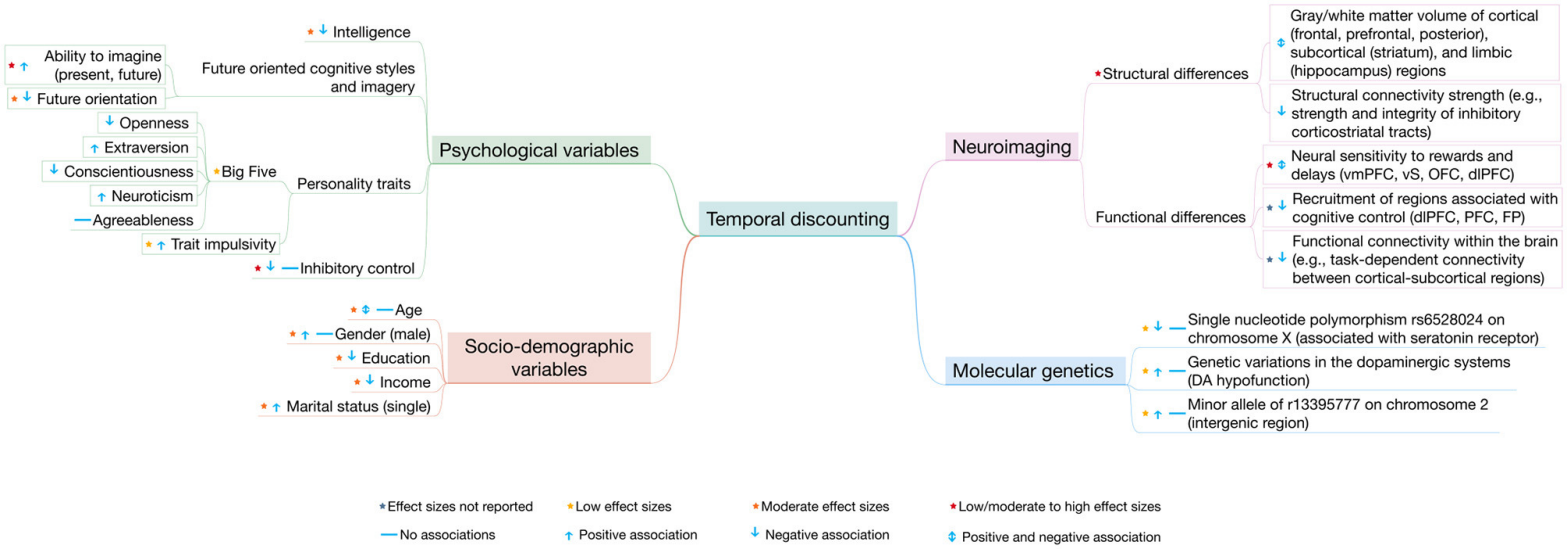
Summary of the associations between temporal discounting and socio-demographic, psychological, neuroimaging, and molecular genetic variables. The figure in a simplified way summarizes how the different variables relate to (steep) temporal discounting. However, the relation of these variables to temporal discounting is far from being so simple and isolated (as it may appear in the figure) as these variables often interact with each other. Low effect sizes are considered *d* (or *r*) < 0.15; moderate effect sizes are considered 0.15 ≤ *d* (or *r*) ≤ 0.35; high effect sizes are considered *d* (or *r*) > 0.35. vmPFC, ventromedial prefrontal cortex; vS, ventral striatum; OFC, orbitofrontal cortex; dlPFC, dorsolateral prefrontal cortex; PFC, prefrontal cortex; FP, frontal pole; DA, dopamine.

Here, we review studies that have begun to shed light on individual differences in temporal discounting and the variables that are associated with it, with a view to elucidating the determinants of observed inter-individual variability and, ultimately, the mechanisms of intertemporal choice. We focus on key individual differences variables, including socio-economic factors, personality, cognition, brain structure and function, as well as molecular genetic variation (Table 1, Figure 2). We critically evaluate these studies and make methodological recommendations for individual differences research in intertemporal choice and decision neuroscience more broadly. Whilst we discuss findings from personality traits with evident clinical relevance such as impulsivity, we stop short of reviewing findings of altered intertemporal choice in mental disorders, as this literature has recently been thoroughly reviewed elsewhere (Amlung et al., 2019).

**Table 1:** Summary of the associations between temporal discounting and socio-demographic, psychological, neuroimaging, and molecular genetic variables.

**Variables**	**Direction of association**	**Effect sizes**
**SOCIO-DEMOGRAPHIC VARIABLES**		
*Age*	No association, Positive, Negative	Moderate
*Gender*	No association, Men discount more	Moderate
*Education level*	Negative	Moderate
*Income*	Negative	Moderate
*Marital status*	Singles discount more	Moderate
***Limitations:*** Small sample sizes, low-powered studies, the correlation between socio-demographic variables and temporal discounting is prone to misinterpretations and should be considered together with other biological and psychological variables		
**PSYCHOLOGICAL VARIABLES**		
*Personality*		
Big Five		
Openness	Negative	Low
Extraversion	Positive	Low
Conscientiousness	Negative	Low
Neuroticism	Positive	Low
Agreeableness	No association	
Trait impulsivity	Positive	Low to moderate
*Future oriented cognitive styles and imagery*		
Ability to imagine (present, future)	Positive	Moderate to high
Future orientation	Negative	Low to moderate
*Intelligence*	Negative	Moderate
*Inhibitory control*	No association, Negative	Low to high
***Limitations:*** Small sample sizes, low correlations, interaction effects are often not considered, effect of possible mediator and moderator variables not sufficiently explored (e.g., the role of risk preferences), impact of task specificities on the obtained results		
**NEUROIMAGING**		
*Brain structure*		
Gray matter volume of regions associated with valuation (vmPFC, striatum, PCC, OFC)	Positive, Negative	Low to moderate
Gray and white matter volume of regions associated with cognitive control (dlPFC, FP)	Positive, Negative	Low to high
White matter volume and cortical thickness of regions associated with memory and future oriented thinking (hippocampus/parahippocampus, entorhinal cortex)	Positive, Negative	Low to high
Connectivity strength of (inhibitory) corticostriatal tracts	Negative	Moderate to high
*Brain function*		
Neural sensitivity to rewards and delays (vmPFC, vS, OFC, dlPFC)	Positive, Negative	Low to high
Recruitment of PFC regions during reward anticipation, valuation, and choice	Negative	Not reported
Intrinsic connectivity strength between cortical regions associated with cognitive control and valuation (e.g., FP and vmPFC)	Negative	Low to moderate
Task-related functional connectivity between cortical and subcortical (e.g., dlPFC and striatum), cortical and limbic (e.g., ACC and hippocampus), and within cortical areas (e.g., dlPFC and vmPFC)	Negative	Not reported
***Limitations:*** Low-powered studies, small sample sizes, inconsistent and non-replicated findings, moderator and mediator variables not thoroughly considered, dependence of the results on the methodology		
**MOLECULAR GENETICS**		
*Genetic variations in the dopaminergic systems associated with DA hypofunction*	No associations, Positive	Low to moderate
*Single nucleotide polymorphism rs6528024 on the X chromosome (associated with internalization of a serotonin receptor)*	No associations, Negative	Low
*Minor allele of r13395777 (intergenic region) on chromosome 2*	No associations, Positive	Low
***Limitations:*** Low-powered studies, non-replicated findings, task impurity problem, no studies on environment × gene interactions		

*Low effect sizes are considered d (or r) < 0.15; moderate effect sizes are considered 0.15 ≤ d (or r) ≤ 0.35; high effect sizes are considered d (or r) > 0.35. vmPFC, ventromedial prefrontal cortex; PCC, posterior cingulate cortex; OFC, orbitofrontal cortex; dlPFC, dorsolateral prefrontal cortex; FP, frontal pole; vS, ventral striatum; ACC, anterior cingulate cortex; DA, dopamine*.

### Measuring Temporal Discounting

Most people prefer a higher reward to a lower reward, ceteris paribus. However, most people also prefer a reward received sooner to a reward of similar magnitude received at a later time. This introduces a trade-off in people's preferences between reward magnitude and the delay until the reward is received. One way to express this trade-off is a utility (or subjective value) function, a function that maps reward magnitude and delay onto units of utility. It is commonly assumed that the utility of a reward *r*_τ_, received after delay τ, is given by:

$$\begin{array}{l} U\left(r_{\tau };\;k\right)=d\left(\tau ;k\right)u\left(r_{\tau }\right) \end{array}$$

where *d* is the so-called discount function with *discount rate k*, reflecting the strength of discounting, and *u* is the utility function. Higher values of *k* indicate stronger discounting (Figure 1).

Utilities can be used to rank rewards of different magnitudes received at different delays. A decision-maker is assumed to be indifferent between two rewards received after different delays if their utilities are the same. In experimental studies of intertemporal choice, the utility function *u* is often assumed to be linear, which means that the utility of a reward is given by its discounted value (Andersen et al., 2008).

A decision-maker's discount rate is not directly observable. It is typically estimated based on a person's choices. A common way to elicit a person's discount rate is an intertemporal choice task, in which a person is asked to make a number of pairwise comparisons between smaller, sooner and larger, later rewards. The smaller, sooner reward is often a fixed amount received immediately (e.g., “$20 now”). An example of such a pairwise comparison might be “$20 now or $30 in 1 month?” (Andersen et al., 2008). It should be noted, however, that specific implementations of intertemporal choice tasks vary, as will be demonstrated throughout the review.

Such tasks can not only be used to measure a person's discount rate but also the functional form of the discount function. Typically, at least three candidate functional forms are considered (Figure 1C): (1) an exponential discount function; (2) a hyperbolic discount function; and (3) a quasi-hyperbolic discount function. The exponential discount function takes the form *d*(τ; *r*) = 1/(1 + *r*)^τ^. The higher the discount rate *r*, the lower the value of the discount function, that is, the more future rewards are discounted (Figure 1B). In the hyperbolic model, the discount function is given by *d*(τ; *k*) = 1/(1 + *kτ*). The latter function is steeper than the exponential function for smaller delays. Another alternative is the quasi-hyperbolic model with discount function *d*(τ; *k*) = βδ^τ^, where β is typically referred to as “present bias,” indicating a premium that decision makers put on immediate rewards (or a discount that they apply to any delayed reward, irrespective of delay), whereas δ captures the discount rate as a function of delay, δ(τ) = 1/(1 + *r*)^τ^.

## Socio-Demographic Factors

Discount rates are associated with a number of fundamental socio-demographic factors. One important factor is age. Whilst temporal discounting decreases from childhood into adolescence and young adulthood (Olson et al., 2007; de Water et al., 2014; Achterberg et al., 2016), the overall evidence about the effects of aging is mixed. While some studies found no differences between older and younger adults (Green et al., 1999; Chao et al., 2009), others have shown either decreased (Meier and Sprenger, 2010; Eppinger et al., 2012) or increased temporal discounting (Read and Read, 2004) in older adults, although there may also be non-linear effects, with discount rates lower in middle age (41–50) compared to younger (19–40) and older (51–75) adults (Harrison et al., 2002). It has been suggested that this inconsistency in age-related differences in temporal discounting may stem from variability in age-related decline of cognitive functions that may contribute to intertemporal choice (James et al., 2015; Huffman et al., 2019), in particular, declarative memory (Kable et al., 2019). Age-related mild-cognitive impairment has been shown to be associated with increased temporal discounting in older people (Kable et al., 2019).

Results of studies of sex differences in temporal discounting are not consistent either (Kirby and Maraković, 1996; Harrison et al., 2002; de Wit et al., 2007; Reimers et al., 2009; Steinberg et al., 2009; Meier and Sprenger, 2010), although a recent meta-analysis argued that men have higher discount rates than women (Gaillard et al., 2020). Lower discount rates are associated with higher educational attainment, higher income and wealth levels, as well as home ownership (Harrison et al., 2002; Meier and Sprenger, 2010; Yang, 2016). Considering marital status, singles appear to have higher discount rates than married people (Harrison et al., 2002).

Correlations of socio-demographic variables with discount rates need to be interpreted with care. An individual's behavioral, and neural phenotype such as temporal discounting at the moment of measurement is the product of a complex developmental process, during which genes, social experience, and cultural context interact with each other dynamically and reciprocally (Rippon et al., 2014). If not properly accounted for in the analysis, associations between discount rates and socio-demographic variables are prone to misinterpretation. Isolating the (causal) effect of individual factors on intertemporal preferences is difficult and requires careful consideration of hypotheses, sample size, independent and dependent variables, as well as type of statistical analysis (Rippon et al., 2014). Unfortunately, many existing studies fall short from a methodological perspective. Large-scale, population-representative studies are needed in order to dissect contributions of different socio-economic as well as other individual differences variables to temporal discounting.

## Personality Traits

Impulsive decision making has been associated with a broad range of personality traits for a long time (Funder et al., 1983; Shoda et al., 1990). *Personality*, defined as characteristic patterns of thought, emotion, and behavior, is a key individual differences variable in psychology. Major theories of personality (Eysenck, 1947; Cloninger, 1986; Gray, 1987; Costa and McCrae, 1992) include dimensions that appear conceptually related to the ability to withhold or delay rewards, such as impulsivity or reward dependence. Accordingly, the most widely studied personality construct in the context of temporal discounting is *trait impulsivity*, often defined as “a predisposition toward rapid, unplanned reactions to internal or external stimuli without regard to the negative consequences of these reactions to the impulsive individual or to others” (Moeller et al., 2001, p. 1784). Within individual differences psychology, impulsivity is considered a broad construct composed of several distinct traits (cf. Whiteside and Lynam, 2001) and is measured with self-report scales such as the UPPS-P Impulsive Behavior Scale (Lynam et al., 2006; Cyders et al., 2014) and the Barratt Impulsiveness Scale (BIS; Patton et al., 1995).

Numerous studies of associations between temporal discounting and impulsivity in relatively small clinical and/or non-clinical samples have reported inconsistent results, ranging from non-significant to moderate correlations between temporal discounting and impulsigenic traits (de Wit et al., 2007; Murphy and MacKillop, 2012; Stahl et al., 2014; Caswell et al., 2015; Steward et al., 2017; VanderBroek-Stice et al., 2017; Jauregi et al., 2018). Recently, a large-scale general population study (*N* = 1,252) indicated that only some impulsigenic traits are significantly related to discount rates (MacKillop et al., 2016). In that study, lack of premeditation and positive urgency from the UPPS-P as well as non-planning and motor impulsivity from the BIS were consistently and significantly related to temporal discounting. However, effect sizes were low (*r* = 0.10) and comparable to those reported in meta-analyses on the relations between self-reported and laboratory measures of impulsivity (Cyders and Coskunpinar, 2011; Sharma et al., 2014). A recent study using genetic data also confirmed weak to moderate positive correlations between trait impulsivity and temporal discounting, with lack of premeditation showing the strongest association (Gustavson et al., 2020). Interestingly, a meta-analysis of the construct of self-control—typically defined as the opposite of impulsivity and often referred to in the context of impulsive decision making (Evenden, 1999)—in part found slightly higher correlations of self- and informant-report questionnaires with temporal discounting, dependent on the concrete temporal discounting task (average *r* = 0.15, range 0.08–0.39 for self-report; average *r* = 0.21, range 0.11–0.27 for informant-report; Duckworth and Kern, 2011).

Thus, although intertemporal choice is sometimes thought to represent a direct behavioral measure of choice impulsivity (Hamilton et al., 2015), associations with traits related to impulsivity are surprisingly low, and often non-significant in underpowered studies (Murphy and MacKillop, 2012; Stahl et al., 2014). It may be suspected that these low correlations are due in part to the heterogeneity of constructs referred to as impulsivity (Evenden, 1999; Strickland and Johnson, 2020) or reliability-related issues in the comparison of self-report and behavioral measures (Enkavi et al., 2019; Dang et al., 2020).

It has to be noted, however, that steeper discounting is generally related to impulsive behaviors such as substance use, gambling behavior, or obesity (Reynolds, 2006; Reimers et al., 2009; see Moreira and Barbosa, 2019, for a recent review) and, accordingly, to pathological behavior (Amlung et al., 2019). Since many personality traits are considered to be on a continuum with psychopathology (Widiger and Samuel, 2005; Coghill and Sonuga-Barke, 2012; Ettinger et al., 2014), these results are not surprising: By definition, some forms of psychopathology involve an extreme form of impulsivity (e.g., ADHD, bulimia nervosa), thus rendering the measurement of this construct very important. Indeed, Amlung et al. (2019) argued that temporal discounting may be a transdiagnostic marker.

Since Whiteside and Lynam (2001) based the original UPPS Scales in part on the Big Five, it has been investigated whether these major dimensions of personality are also related to temporal discounting. The strongest association appears to be between steeper discount rates and higher levels of extraversion (Daugherty and Brase, 2010; Mahalingam et al., 2014; Civai et al., 2016), perhaps due to higher susceptibility to (direct) rewards in extraverted individuals (Ostaszewski, 1996; Hirsh et al., 2010). Similar to impulsivity, however, this association is of small effect: In a study of *N* = 5,888 participants, Mahalingam et al. (2014) found a correlation of *r* = 0.10. Their results also revealed small effects of openness (*r* = −0.05), conscientiousness (*r* = −0.09), and neuroticism (*r* = 0.09), with higher discounting being predicted by lower openness and conscientiousness and higher neuroticism. Agreeableness was not related to temporal discounting. Interestingly, the influences of openness and neuroticism interacted with the magnitude effect (i.e., the effect that smaller rewards are discounted more strongly than larger ones), reflecting the fact that correlations were stronger for larger reward magnitudes (Mahalingam et al., 2014). However, as Mahalingam et al. (2014) point out themselves, the study only used two different delayed amounts and a single delay interval, thereby limiting the psychometric quality of the temporal discounting measure.

Apart from impulsivity and the Big Five, research into associations between temporal discounting and personality traits is scarce and less consistent. For instance, associations between steeper discounting and novelty seeking (Anokhin et al., 2011; Malesza and Ostaszewski, 2013), reward dependency (Malesza and Ostaszewski, 2013), or risk-relevant traits such as harm avoidance (Bobova et al., 2009; Malesza and Ostaszewski, 2013; Howe and Finn, 2020) and sensation seeking (Ostaszewski, 1996; Kirby and Petry, 2004; Stahl et al., 2014) remain unclear.

Notably, interactions between different personality traits such as harm avoidance and impulsivity (Howe and Finn, 2020) were recently highlighted and should be investigated in future studies. This notion is particularly important, given the observation that risk perception, which may to some extent be reflected by harm avoidance, plays an influential role in temporal discounting (Lopez-Guzman et al., 2018). Indeed, it is important to consider that delayed rewards, especially real-life rewards, are also *uncertain*. This may be especially true in socioeconomically-stressed populations (see Socio-demographic factors section) and possibly also moderates associations with various personality traits. Since models without consideration of risk attitudes overestimate discount rates of risk-averse individuals and underestimate discount rates of risk-seeking individuals (Lopez-Guzman et al., 2018), effect sizes of associations between temporal discounting and personality traits might be biased.

Additionally, different methods and measures of temporal discounting may lead to results varying between studies. For instance, both temporal discounting and the possible confounding effect of risk perception could be differentially influenced by different task parameters such as reward method (e.g., hypothetical vs. real rewards; though see Johnson and Bickel, 2002; Matusiewicz et al., 2013), reward magnitude (Prelec and Loewenstein, 1991), or trust toward the experimenter (Ma et al., 2018). For example, the association of temporal discounting with neuroticism might be particularly high when participants are confronted with real rewards, high reward magnitudes, and less trusted experimenters or online settings, partly due to confounds with probability discounting.

Therefore, risk perception factors should be accounted for (Lopez-Guzman et al., 2018, 2019) and the specific implementation of intertemporal choice tasks should be considered when investigating temporal discounting and its association with individual difference variables.

Overall, studies of the personality correlates of temporal discounting have yielded patterns that broadly agree with the construct validity of temporal discounting as measure of impulsivity. However, studies were not entirely consistent, and effect sizes tend to be small. More large-scale studies are required to both test specific hypotheses and replicate previous findings (possibly using the same vs. different temporal discounting applications), whilst also accounting for the possible role of individual differences in risk perception.

## Future-Oriented Cognitive Styles and Imagery

Another important domain of individual differences concerns future-oriented cognitive styles and abilities, i.e., general temporal thinking (Zimbardo and Boyd, 1999) or mental imagery, which is defined as the ability to form and experience mental representations of stimuli without actually perceiving them (Pearson et al., 2015). Since both constructs involve subjective thinking styles and experiences, they are normally assessed with self-report questionnaires. Measures of temporal thinking include the Consideration of Future Consequences Scale (CFCS; Strathman et al., 1994) and the Zimbardo Time Perspective Inventory (ZTPI; Zimbardo and Boyd, 1999), whereas (visual) mental imagery is generally assessed with the Vividness of Visual Imagery Questionnaire (VVIQ; Marks, 1973).

Initially, it may appear straightforward that dispositions that emphasize thinking about the future should be positively associated with choosing larger future rewards over smaller immediate rewards. And indeed, as Teuscher and Mitchell (2011) observed, future orientation and discount rates are similarly related to behavioral and demographic variables and show significant associations with each other, with more future oriented individuals showing less temporal discounting. However, Teuscher and Mitchell (2011) also concluded that the correlation between the two constructs is only modest and that they are not identical. Most importantly, Steinberg et al. (2009), who investigated 935 individuals using a new scale of future orientation, only found a correlation of *r* = −0.15. Later studies assessing other and more widely used measures of future orientation in large samples found comparable effect sizes (Daugherty and Brase, 2010; Bruderer Enzler, 2015; Cosenza and Nigro, 2015).

Overall, although the term future orientation has been used in the context of many different constructs (Steinberg et al., 2009), the correlation of temporal discounting with measures of time perspective appears to be robust, consistently indicating that steeper temporal discounting is associated with stronger focus on present rather than future consequences (Daugherty and Brase, 2010; Guo et al., 2017; Macaskill et al., 2019). Recently, a study replicated these results but raised the question of whether temporal discounting is more strongly related to immediate than future orientation (Macaskill et al., 2019). The authors concluded that studies with relatively elaborate temporal discounting paradigms point to that finding (e.g., Charlton et al., 2011, Study 2; Joireman et al., 2008, Study 3). However, there were also studies finding comparable correlations (with even more participants) that used rather complex tasks as well (e.g., Cosenza and Nigro, 2015).

Thus, although future studies should investigate relations of temporal discounting with specific temporal thinking styles further, it seems that both future and present time orientation have an impact on temporal discounting, perhaps comparable in size. Macaskill et al. (2019) make an important, more general point, though, in emphasizing the impact of task complexity. While shorter temporal discounting tasks may entail an economic advantage enabling large-scale studies (e.g., single-shot paradigms; see Macaskill et al., 2019, Study 1), investigators should bear in mind that this comes at the expense of psychometric quality and might influence associations with individual difference variables.

Apart from thinking styles, the ability to imagine future events has been considered as a correlate of temporal discounting. Since *episodic future thinking*, i.e., making use of episodic memory to pre-experience future events (Atance and O'Neill, 2001), reveals that discounting can be reduced by inducing episodic imagery (Rung and Madden, 2018; Scholten et al., 2019), it is reasonable to assume that interindividual differences in the tendency and ability to imagine are related to temporal discounting. However, Parthasarathi et al. (2017) unexpectedly found that higher ability to imagine (measured using VVIQ, Marks, 1973; lower scores representing more vivid imagery) was associated with stronger temporal discounting (primary training study: *N*_before_training_ = 48, *r*_before_training_ = −0.37; *N*_after_training_ = 38, *r*_after_training_ = −0.45; additional study: *N* = 154, *r* = −0.25). Moreover, a 4-week visualization training, compared to a relaxation training control group, surprisingly led to a (weak) increase in discount rates (*N* = 38; *d*_after_training_ = 0.71)^1^.

It is unclear, however, why these effects occurred. Parthasarathi et al. (2017) provided several possible explanations, including the possibilities that the VVIQ is primarily related to present imagination; that imagery ability could also lead to an enhanced visualization of smaller, sooner options, thus providing an enhanced preference for them; that visualizing isolated future goals reduces motivation toward them. Furthermore, the above-mentioned risk preference issue might also have an influence here, as imagery ability has been shown to moderate the relationship between risk perception and risk taking (Traczyk et al., 2015), thus possibly leading to less influence of risk perception on discounting behavior and, ultimately, more discounting in people with stronger imagery ability. Also, methodological issues such as the dissociation of hypothetical and real rewards should be considered since the ability to imagine the receipt of money now or in future may play a differential role (e.g., a stronger role in hypothetical rewards because it might be generally harder to imagine receipt of pretend money or a stronger role in real rewards because it is more relevant to imagine receipt of real money). However, to date, no studies have been carried out to clarify these important open questions. Thus, future investigations should aim to replicate the effect found by Parthasarathi et al. (2017) and to give evidence for potential explanations. A promising population within which to address these questions consists of participants with extremely low or high voluntary imagery (aphantasics and hyperphantasics, respectively; Zeman et al., 2020). Moreover, investigating interactions between episodic future thinking and self-report measures of imagery could help to determine the role of imagination abilities in temporal discounting.

## Inhibitory Control

*Inhibitory control* refers to the ability to withhold or stop responses or thoughts that are unwanted in a given context. Within the influential model by Miyake et al. (2000), inhibitory control is a core dimension of cognitive control. *Cognitive control*, or *executive function*, refers to effortful, general-purpose control mechanisms that dynamically regulate thoughts and behaviors by modulating cognitive sub-processes (Miyake and Friedman, 2012; Diamond, 2013; Cohen, 2017). Cognitive control is required when automatic or impulsive responses have to be overridden. This ability is of relevance to mental and physical health, success in education, and other real-life outcomes (Egner, 2017). Inhibitory control itself is heterogeneous, but has been suggested to include response inhibition, resistance to distractor interference, and resistance to proactive interference from irrelevant memories (Friedman and Miyake, 2004).

Controlled inhibition of automatic processes and responses may also be a mechanism of intertemporal choice (Cohen, 2017). Accordingly, relationships between temporal discounting and inhibitory control have been examined in both humans and rodents (Dalley et al., 2011). Findings, however, are so far mostly inconclusive.

Urošević et al. (2016) found no significant correlations between temporal discounting and inhibitory control in a go/nogo task in healthy controls (*N* = 32), although a correlation emerged in bipolar patients (*r* = 0.40). A study of *N* = 167 students also failed to observe significant correlations of temporal discounting with go/nogo and stop-signal task performance (Jauregi et al., 2018).

There is, however, also evidence of significant associations. In a comprehensive analysis of impulsivity and inhibitory control in *N* = 190 healthy adults, Stahl et al. (2014) observed that a latent variable indexed by two temporal discounting tasks was correlated with a latent variable tapping inhibition of proactive interference. There was no correlation, however, with latent variables tapping stimulus interference, response interference, and information sampling.

Additionally, Lawyer and Mahoney (2018) found the individual rate of discounting (*b*) to be correlated modestly but significantly with the stop signal reaction time (SSRT) measure from the stop-signal task in *N* = 296 young adults. SSRT was not, however, correlated with another measure of temporal discounting, the area under the curve.

Moreover, Harden et al. (2017) observed in a large sample of *N* = 810 adolescents that temporal discounting loaded onto a “cognitive dyscontrol” factor that included, amongst others, the Tower of London task, a measure of general executive function. This suggests that associations between temporal discounting and aspects of cognitive control may exist, but that they may be of small magnitude and may benefit from latent factor approaches.

Overall, evidence of correlations between temporal discounting and inhibitory control is scant and relatively inconsistent. It should be noted that the lack of strong associations between temporal discounting and cognitive control is complemented by experimental data, which show that these seemingly related constructs do indeed reflect different processes (Scherbaum et al., 2018). Further work is thus clearly needed, particularly with larger samples and employing sophisticated data analytical techniques (Harden et al., 2017).

Future studies may also further explore this area by going beyond studying reaction times and error rates of inhibitory tasks, instead employing model parameters derived from models of task performance (e.g., Ulrich et al., 2015; Aponte et al., 2017) and relating these to temporal discounting parameters (Kvam et al., 2020).

## Intelligence

Intelligence has often been investigated in relation to intertemporal choice. Mischel, the pioneer in the study of delay of gratification, and his colleagues reported that results of their marshmallow test of self-control covary with IQ (Mischel and Metzner, 1962) and longitudinally predict a broad range of competencies (Mischel et al., 1988; Shoda et al., 1990; though see Watts et al., 2018, for possible limitations). Interestingly, in the study of children's tendency to discount the value of a relatively more worthful candy (Mischel and Metzner, 1962), there was a positive correlation between self-control and intelligence (*r* = 0.29), comparable to the effect sizes found in more recent studies of more complex temporal discounting tasks in adults (Shamosh and Gray, 2008).

Indeed, Shamosh and Gray (2008) conducted a meta-analysis of 24 studies and found that higher intelligence was associated with lower temporal discounting (weighted mean using a random effects model: *r* = −0.23). They further found this effect to be independent of the type of temporal discounting measure, experimental paradigm, and domain of intelligence, although several other moderator variables may play a role. For instance, the relation between intelligence and temporal discounting was weaker when real monetary outcomes involved chance (i.e., when participants had a chance of winning a real reward or when one single trial was randomly chosen to assign a real reward to participants) compared to studies in which outcomes were continuously hypothetical or real. Furthermore, measures of verbal intelligence were correlated equally strongly with temporal discounting as combined measures of verbal and non-verbal intelligence, suggesting either a role of verbal strategies to maintain self-control (Olson et al., 2007) or influences of a general intelligence factor (Isen et al., 2014). Finally, the authors explored possible moderating variables such as publication year and sample characteristics [age, education level, socio-economic status (SES), and IQ]. The results suggest that the relationship between temporal discounting and intelligence was stronger in more recent publications as well as in older, more highly educated, and more intelligent samples and those with higher SES. It should be noted, however, that, given the relatively low number of studies involved in the meta-analysis, the exploratory nature of the majority of the moderator analysis, the liberal modeling methods, and the collinearity between some of the variables (e.g., age and education), the differential roles of various background and moderating variables have to be examined further.

Regarding the basis of the observed correlation, Shamosh and Gray (2008) argued that it might be the result of either motivational processes, such as conscientiousness, or shared mechanisms, such as working memory (Hinson et al., 2003). Age-related cognitive decline may also play a moderating role here (Kable et al., 2019). Generally, studies point to a combination of several variables: For instance, since the relation between the Big Five and temporal discounting is significant but rather low (see Personality Traits section), it can be assumed to be of some relevance (Civai et al., 2016), but additional variables should be considered.

Shamosh et al. (2008) tested the hypothesis of shared working memory mechanisms, as represented by working memory capacity, which is the ability to actively maintain goal-relevant mental representations (such as future rewards), while suppressing irrelevant or competing information (such as immediate rewards; Engle, 2002, 2018). They computed a latent variable derived from four working memory span tasks and a three-back task and measured brain activity with fMRI. Behaviorally, controlling for intelligence, working memory had no unique contribution to temporal discounting. Moreover, the relation between intelligence and temporal discounting was partially mediated by working memory-related brain activity in the left anterior prefrontal cortex (aPFC), thereby confirming the hypothesis of shared working memory mechanisms but also indicating the relevance of other variables, such as demographic variables and levels of future orientation. In agreement with these findings, it has been discussed that, apart from higher executive control functions, more precise perception of future outcomes could be relevant for the observed association (Burks et al., 2009)—an assumption that also is related to working memory and future orientation (see sections above). In fact, Basile and Toplak (2015) revealed in *N* = 99 that executive function and dispositions to think toward the future together are able to render intelligence non-significant as predictor of temporal discounting. Furthermore, a recent study demonstrated that working memory training could possibly lead to lower discount rates (Felton et al., 2019; also see Rung and Madden, 2018; Scholten et al., 2019). In sum, executive functioning seems to be an important underlying process relating intelligence and temporal discounting, and a disposition toward future orientation could be relevant as well. However, to the best of our knowledge, to date, no large-scale study has compared several moderating or mediating factors to completely explain the correlation.

Since the meta-analysis by Shamosh and Gray (2008), numerous studies on the relation between temporal discounting and intelligence have been added to the literature. Amongst them are large-scale studies that, though addressing different primary research aims, generally confirm the significant association of temporal discounting and intelligence found by Shamosh and Gray (2008) with similar effect sizes (e.g., Isen et al., 2014: *r* = |0.17|–|0.28|; Steinberg et al., 2009: *r* = |0.27|–|0.31|). Based on the groundbreaking investigations by Mischel and colleagues and studies in later years (Isen et al., 2014), the association appears to be stable over the life course, suggesting that both intelligence and the tendency to choose larger, later over smaller, sooner rewards develop similarly. In fact, both could be represented in neural development, notably within PFC, again suggesting a role of executive functions (Shamosh et al., 2008; Wesley and Bickel, 2014). However, as mentioned above, further—large-scale or possibly meta-analytic—studies on the relation between temporal discounting and intelligence are needed to clarify the role of different measures of intelligence as well as of shared underlying processes and moderating variables.

## Brain Structure and Function

Associations of temporal discounting with brain structure and function may to some extent explain links between individual differences in temporal discounting and other psychological variables. In this section, we review neuroimaging evidence that relates variation in brain structure, activity, and connectivity patterns with individual differences in temporal discounting. It is important to note that our aim is not to thoroughly review the neural underpinnings of temporal discounting; this has been done in previous work (Peters and Büchel, 2011; Frost and McNaughton, 2017; Schüller et al., 2019).

Evidence from structural imaging studies suggests that regional gray (gMV) and white matter volumes can to some extent predict individual differences in discount rates. In general, temporal discounting correlates with (i) gMV of regions associated with valuation such as striatum, ventromedial prefrontal cortex (vmPFC), posterior cingulate cortex (PCC), and orbitofrontal cortex (OFC) (Cho et al., 2013; Tschernegg et al., 2015; Guo et al., 2017), (ii) gMV of regions associated with cognitive control and other more deliberate processes such as dorsolateral prefrontal cortex (dlPFC) and frontal pole (FP) (Bjork et al., 2009; Mohammadi et al., 2016; Wang et al., 2016), as well as (iii) gMV and white matter volume of regions associated with memory and future-oriented thinking (hippocampus/parahippocampus, entorhinal cortex; Yu, 2012; Wang et al., 2016; Lempert et al., 2020).

However, not only the strength, but also the direction of the reported correlations is inconsistent across studies. For instance, in *N* = 34 healthy individuals, Cho et al. (2013) found that discount rates correlate negatively with gMV of striatum (bilateral putamen, no correlation coefficient reported). On the other hand, employing a region of interest (ROI) approach, using a more detailed assessment of temporal discounting (more items, repeated testing), and a larger sample size (*N* = 70), Tschernegg et al. (2015) found a positive correlation between striatal gMV and discount rate (reported correlations for left caudate: *r* = 0.28, *p* = 0.02, uncorrected; and for right caudate: *r* = 0.31, *p* = 0.01, uncorrected). Similarly, while Bjork et al. (2009) (*N* = 29) reported negative correlations between gMV of regions associated with cognitive control and discount rate (inferior lateral PFC: β = −0.37, *p* < 0.05, uncorrected; dlPFC: β = −0.42, *p* < 0.05, uncorrected), Wang et al. (2016) (*N* = 227) demonstrated that gMV of similar regions (FP, OFC, lateral PFC) correlates positively with discount rate (prediction accuracy rates ranging from 0.17 to 0.24). These inconsistent findings may be attributed to several factors including sample heterogeneity across studies, differences in approach (whole brain vs. ROI), methods to assess temporal discounting, statistical analyses (e.g., differences in the chosen thresholds), and the anatomic specificity of the reported brain regions.

Individual differences in temporal discounting may also be related to task-dependent neural activity differences (Seghier and Price, 2018). Specifically, it has been argued that the degree of PFC recruitment during temporal discounting may among others reflect differences in self-control and thus relate to temporal discounting. Supporting this, it has been shown that increased activity in cognitive control associated regions during temporal discounting relates to choosing larger, later rewards, whereas hypofunction in these areas is associated with impulsive choice (McClure et al., 2004; Peters and Büchel, 2011; Elton et al., 2017; Jimura et al., 2018).

Other studies have shown that individual differences in neural sensitivity to temporal delays and reward magnitudes may relate to differences in temporal discounting (Hariri et al., 2006; Kable and Glimcher, 2007; Ballard and Knutson, 2009; Cooper et al., 2013). Cooper et al. (2013) reported that steep and flat discounters exhibit opposite neural patterns in response to delays; while the former showed increased activity in ventral striatum (vS) and vmPFC in response to short delays, this pattern was observed for long delays in the latter. Furthermore, the authors demonstrated that neural delay sensitivity could account for ~15% of variance in discount rates. This value is close to the reliable correlation between cognitive ability and temporal discounting (Shamosh and Gray, 2008), thereby supporting a significant contribution of neural delay sensitivity to differences in temporal discounting. Differences in vS sensitivity to rewards have also been implicated in temporal discounting. However, the exact nature of this association in healthy adults remains debatable (Hariri et al., 2006; Ballard and Knutson, 2009; de Water et al., 2017; Elton et al., 2017).

In addition to the sensitivity and recruitment of certain brain regions, the effectiveness of the structural, intrinsic, and functional connectivity between and within these regions has been shown to predict differences in temporal discounting (Li et al., 2013; van den Bos et al., 2014; Calluso et al., 2015; Mohammadi et al., 2016; Wang et al., 2016; Anandakumar et al., 2018; Ikuta et al., 2018; Xin et al., 2020). van den Bos et al. (2014) demonstrated that individual differences in discount rates were correlated with structural and functional connectivity between right dlPFC and dorsal striatum (*r* = −0.66, *p* < 0.001 and *r* = 0.57, *p* < 0.01 respectively; *N* = 22). More specifically, they found that stronger structural corticostriatal connectivity and stronger negative corticostriatal functional coupling during temporal discounting predict lower discount rates. Similarly, flat discounters have been found to exhibit stronger intrinsic (Wang et al., 2016) and task-dependent functional connectivity between cortical and subcortical, cortical and limbic (Peters and Büchel, 2010, 2011), and within cortical areas (Hare et al., 2014), than steep discounters. Considering that temporal discounting behavior is complex and engages several brain networks, future research should more precisely investigate how different neural networks interact during temporal discounting behavior, and whether inter-individual differences in these network interactions relate to differences in temporal discounting.

Brain features may also explain or mediate the link between psychological variables (such as personality traits and cognition) and temporal discounting. For instance, Shamosh et al. (2008) showed that working memory-related aPFC activity partially mediates the relationship between intelligence and temporal discounting (see Intelligence section), whereas Benoit et al. (2011) showed that activity in middle rostral PFC mediates the relation between temporal discounting and episodic future thinking. Relatedly, resting state functional connectivity (Guo and Feng, 2015) and intrinsic organization within PFC (Xin et al., 2020) have been shown to support the relation between temporal discounting and personality dispositions such as regulatory mode (Guo and Feng, 2015) and achievement motivation (Xin et al., 2020), respectively. Anterior and lateral portions of PFC have been associated with information integration (anterior/rostral portions of PFC; Ramnani and Owen, 2004) and executive functions of working memory and attention (lateral portions of PFC; Yuan and Raz, 2014). Thus, it could be speculated that differences in processes such as memory, attention, and information integration may underlie differences in temporal discounting. More powerful studies are needed to replicate these findings and to assess the possible mediating mechanisms underlying the correlations between temporal discounting and other psychological variables.

Taken together, neuroimaging studies provide evidence that structures and functional patterns in prefrontal, subcortical, and limbic regions associated with valuation, cognitive control, memory, and future-oriented thinking may partially account for individual differences in temporal discounting. However, more research is needed to replicate and validate the findings in large, representative samples.

These results should be interpreted with caution for several reasons. First and foremost, the results are not conclusive and are often derived from relatively low-powered studies. The correlation strength (effect size) between MRI measures (both structural and functional) and individual differences is typically very small, which renders small sample studies severely underpowered and prone to false negatives. This is especially the case for fMRI studies (the studies reviewed here have sample sizes ranging from *N* = 12 to *N* = 103), as more recent structural studies on temporal discounting have used relatively larger sample sizes (e.g., Wang et al., 2016, *N* = 227). Considering the low correlation between MRI measures and individual differences in temporal discounting, the inconsistent findings reported in this section, and more generally the replicability crisis in psychological research, future neuroimaging studies should use larger sample sizes to increase statistical power (Turner et al., 2018). In addition, larger sample sizes will make it possible to more thoroughly consider the effects of moderator or mediator variables such as age, sex, IQ, or SES and thus better discern the contribution of each to differences in temporal discounting behavior.

Second, a recent meta-analysis (Elliott et al., 2020) reported low test–retest reliability of commonly used fMRI measures, which hinders and limits the ability to use task-fMRI measures for individual differences research. While temporal discounting tasks were not included in the meta-analysis, previous research indicates that they may not be immune to reliability issues (Vetter et al., 2017; Fröhner et al., 2019). Therefore, unless evidence of reliability is provided, task-fMRI studies linking individual differences in temporal discounting with brain features should be considered with great caution.

Third, findings appear to depend on approach and methodology. One example concerns differences when employing whole brain vs. ROI approaches. While the former may provide a broader overview of the brain regions associated with temporal discounting behavior, the latter may offer insights into the role of more specific brain regions, alas with additional limitations stemming from methods used to define specific ROIs. Moreover, recent work (Botvinik-Nezer et al., 2020) indicates that even within the same approach, differences in analysis pipelines may yield different findings. Therefore, combining different approaches and employing meta-analytic strategies may provide a better picture of the neural correlates underlying individual differences in temporal discounting.

Finally, many neuroimaging studies do not report effect sizes, making it difficult to compare findings across studies and to quantify the magnitude of the reported effects. To aid cross-study comparisons and to aid meta-analyses, future studies should support their conclusions with effect sizes [e.g., for fMRI studies, Poldrack et al. (2008) suggest mean % signal change and standard deviation].

## Molecular Genetics

Converging evidence from animal (Anderson and Woolverton, 2005; Stein et al., 2012) and human studies (Isen et al., 2014; Anokhin et al., 2015) shows that temporal discounting is moderately yet significantly heritable. Building on this finding, several genetic polymorphisms have been associated with temporal discounting; however, the findings are inconclusive (Mitchell, 2011; MacKillop, 2013; Gray et al., 2019; Levitt et al., 2020).

Given the involvement of the neuromodulator dopamine (DA) in motivation and reward processing (Schultz, 2001) as well as clinical conditions associated with steep discounting (e.g., addiction; Le Folla et al., 2009; Mitchell, 2011), several studies have investigated how variations in genes coding for the synthesis, signaling, re-uptake, and metabolism of DA relate to temporal discounting (Boettiger et al., 2007; Eisenberg et al., 2007; Paloyelis et al., 2010; Sweitzer et al., 2013; MacKillop et al., 2015). Although these candidate-gene studies converge on the importance of the DA system in temporal discounting (Gray et al., 2019; Levitt et al., 2020), the results as to which genes and which variations relate to steeper discounting are largely inconsistent and have not been replicated in other studies. A major reason for this is the insufficient power of these studies to detect small effects (most probable in this case), which severely compromises the reliability and validity of the findings^2^.

Currently, the most powerful approach in molecular genetics is the Genome Wide Association Study (GWAS) design. To date, there are two GWAS of temporal discounting (Sanchez-Roige et al., 2018; MacKillop et al., 2019). In the largest of these (Sanchez-Roige et al., 2018), steeper discounting was associated with a polymorphism (rs6528024) in the *GPM6B* gene (genome-wide significance *p* = 2.40 × 10^−8^, β = −0.10, SEM = 0.02, minor allele frequency = 0.03), which is involved in the internalization of a serotonin receptor. Importantly, these results were replicated in an independent cohort (*N* = 928; *p* = 1.44 × 10^−8^, β = −0.10, SEM = 0.02). In that study, the authors additionally showed that 12% of the variance in temporal discounting was accounted by genotype. By contrast, in a more recent GWAS in *N* = 986 healthy individuals, MacKillop et al. (2019) found an association between temporal discounting and a polymorphism (rs521674) in the *ADRA2A* gene coding for α2 adrenoreceptor (which did not survive correction for type I error rate), and an intergenic locus in chromosome 2 (genome-wide significant at *p* = 2.8 × 10^−8^, β = 0.27, SEM = 0.05, minor allele frequency = 0.33), whose function remains unknown. Interestingly, in addition to not reporting concurrent findings, these studies did not replicate previous findings from candidate-gene approaches.

Taken together, these findings suggest that the genetic underpinnings of temporal discounting are far more complex than may have been initially thought. Further research is needed to replicate these findings in well-powered studies with appropriate replication samples. Multi-center studies and large-scale consortia may be needed to achieve these goals.

Similar to brain features, genetic variations may explain, mediate, or moderate correlations between temporal discounting and different psychological variables. In line with this, Sanchez-Roige et al. (2018) found that the genetic signature of temporal discounting overlapped with neuroticism (*r*_g_ = 0.18, *p* = 2.25 × 10^−2^), years of education (*r*_g_ = −0.67, *p* = 7.9 × 10^−15^), childhood IQ (*r*_g_ = −0.63, *p* = 1.63 × 10^−4^), and college attainment (*r*_g_ = −0.93, *p* = 3 × 10^−10^), all of which have also been behaviorally associated with temporal discounting (see above). These findings have yet to be replicated in more powerful studies. A promising approach that could additionally complement these findings is using GWAS to investigate whether psychological variables and temporal discounting share common genetic variations. This approach has been used to assess the genetical relationship between major psychiatric disorders (Lee et al., 2013), and may provide additional evidence on the biological mechanisms underlying associations between psychological variables and temporal discounting.

Several considerations should be taken into account in this context. First, evidence from behavioral genetics (Anokhin et al., 2011, 2015; Isen et al., 2014) confirms that temporal discounting is not completely genetically determined. Moreover, other factors may mediate and/or moderate the molecular genetic effects reviewed here. For instance, Gianotti et al. (2012) demonstrated that baseline activity in PFC mediated the association between Catechol-*O*-Methyltransferase (*COMT*) Val158Met polymorphism and temporal discounting. These and similar findings could point to a potential “third variable problem,” considering that the same genes and the same areas are associated with other variables such as for example working memory (in the case of PFC activity, see Lara and Wallis, 2015). Therefore, it is imperative to employ more integrative approaches, whereby several influences are considered together such that genetic contributions to temporal discounting can be more clearly defined.

Second, an important aspect in this line of research is employing a framework which will help in understanding how molecular genetic variation, temporal discounting, and psychiatric illness phenotypes co-occur. An increasingly promising framework proposes that temporal discounting may play the role of an endophenotype, viz. a marker of the genetic liability for a clinical disorder (Gottesman and Shields, 1973; Gottesman and Gould, 2003). While discussing the endophenotypic nature of temporal discounting is beyond the scope of this review (for a review see MacKillop, 2013), it is important to highlight that understanding the genetic underpinning of temporal discounting in healthy individuals may also provide invaluable insights in further elucidating the endophenotypic nature of temporal discounting and, by extension, the etiology, and pathophysiology of major clinical disorders (Amlung et al., 2019).

Finally, as discussed throughout this review, temporal discounting is a complex behavior determined by several genetical and environmental factors. Surprisingly, however, interactions between genes and environment in temporal discounting remain unexplored.

Overall, the methodological shortcomings, inconsistencies and gaps in the literature discussed here should inspire future research to employ more integrative and innovative approaches that could ultimately paint a more comprehensive picture of temporal discounting.

## Discussion

We have provided a comprehensive narrative review of temporal discounting from an individual differences perspective. Here, we (i) briefly summarize the key findings and (ii) discuss both limitations of previous studies and implications for future research (Table 1, Figure 2).

### Summary of Key Findings

The first key finding is that temporal discounting, despite its ubiquity in human and non-human animals alike, is subject to considerable individual differences; i.e., people generally discount future rewards, but they vary in the extent to which they do so. Importantly, this variation is not due to random fluctuations but is both temporally stable and significantly heritable.

Second, individual differences in temporal discounting are associated with a number of socio-economic and health-related variables. Where replicable findings are observed, the direction of these associations is unambiguous: Steeper discount rates are associated with less economic success and greater levels of health problems.

Third, large studies within the domain of personality have shown that steeper temporal discounting is associated with higher levels of self-reported impulsivity and extraversion; however, effect sizes are small. Steeper discounting may also be related to lower openness and conscientiousness as well as higher neuroticism. Relations of temporal discounting with constructs such as novelty-seeking, reward dependence, harm avoidance, or sensation seeking are less consistent.

Fourth, cognitive styles related to future orientation show overlap with temporal discounting in studies of large samples, again with small effect size. Investigations of the relation between temporal discounting and the ability to imagine future events are scarce and have not yielded conclusive results yet.

Fifth, not many studies have investigated correlations between temporal discounting and inhibitory control, and findings are not entirely consistent. However, large studies point to overlaps of small magnitude between temporal discounting and facets of inhibitory control.

Sixth, there is consistent evidence of an association between steeper temporal discounting and lower intelligence. The strength of the association appears greater than those with personality or cognitive variables and is robust regarding various measurement features. However, likely moderator variables include age, education, sample's level of intelligence, and SES.

Seventh, individual differences in brain structure and function have been related to temporal discounting in a growing number of studies. However, findings are as yet not consistent, both with regard to areas involved and direction of effects.

Finally, following early candidate gene studies, more recent work has used GWAS. Findings are inconsistent and suggest that the molecular genetic architecture of temporal discounting is more complex than initially thought.

### Limitations and Implications

The above reviewed body of work has produced many important and replicable findings; however, no studies or research fields are without limitations. The limitations of these studies will now be discussed, combined with suggestions for future research.

First, significant test–retest reliabilities have been interpreted as evidence that variance in temporal discounting has a trait component. However, no study has formally modeled the state and trait components underlying variance in temporal discounting. These can be separated from each other and from measurement error using statistical techniques derived from latent state-trait (LST) theory, which allow the estimation of amounts of variance in a measurement due to stable trait, situational fluctuations (state) and person × situation interactions, and measurement error (Geiser et al., 2015). LST modeling of temporal discounting may inform further individual differences studies with regards to the amount of variation in temporal discounting that is in fact due to traits rather than states or measurement error.

Second, despite replicable correlations between socio-demographic variables and discount rates, their origins are unclear. An area of particular need of further investigation concerns the vexing issue of the direction of effects, i.e., whether steeper temporal discounting causes socio-economic disadvantage or vice versa. Combined cross-sectional and longitudinal designs are needed to disentangle these relationships.

Third, a take-home message of work in personality is that correlations with temporal discounting are of small magnitude, often approximately *r* = 0.10 (Mahalingam et al., 2014; MacKillop et al., 2016). An implication, which cannot be stressed enough, is that large samples are needed to detect such effects with high confidence. To provide a simple guideline, an a priori power calculation (Faul et al., 2009) for a correlation with an expected effect size of rho = 0.10, alpha level of 0.05 (one-tailed) and power of 0.90 yields a required sample size of *N* = 850. To obtain 0.95 power, a sample size of *N* = 1,073 is needed. While some studies fulfill these demands, many in the field do not. Accordingly, findings from smaller studies are often inconsistent or negative, and the likely robust estimates of small correlations come from large samples (e.g., Steinberg et al., 2009; Mahalingam et al., 2014; MacKillop et al., 2016; Harden et al., 2017).

A further suggestion for personality research is that future studies should be clear about whether correlations were planned a priori and hypothesis-driven or exploratory. Both approaches have their place (Behrens, 1997; Wagenmakers et al., 2012). However, not only given that psychometric personality questionnaires can be easily administered and yield numerous variables, pre-registered (replication) studies are sorely needed to consolidate and advance the field.

A fundamental issue concerns the important question of why correlations are so low. This particularly concerns psychometric self-report measures of impulsivity given that temporal discounting has often been considered a measure of impulsivity (Padhi et al., 2012; Patton and Stanford, 2012). The problem is, however, not unique to intertemporal choice. For example, correlations between psychometric self-report measures of impulsivity on the one hand and task-based measures of inhibitory control on the other hand tend to be similar in magnitude to those observed here (approximately 0.10–0.15; Cyders and Coskunpinar, 2011; Aichert et al., 2012). A number of reasons have been proposed for this phenomenon, relating both to methodological and theoretical issues. A methodological criticism that has been made of task-based measures is their low reliability (Hedge et al., 2018), which puts an upper limit on the correlation that can be observed with a psychometric self-report measure. In the case of temporal discounting, that criticism is not likely to play a major role, given that temporal discounting shows good-to-high test–retest reliabilities (Beck and Triplett, 2009; Kirby, 2009; Jimura et al., 2011; Matusiewicz et al., 2013; Martínez-Loredo et al., 2017). A more substantial, theoretical criticism relates to the constructs themselves (Dang et al., 2020). Specifically, even though two measures may share the same name, they may not measure the same construct (a problem known as the jingle-jangle fallacy; Marsh, 1994). This is particularly pertinent for constructs as heterogeneous as impulsivity. Thus, correlations between temporal discounting and psychometric self-report measures of impulsivity may be low, because they tap different aspects of the broad construct of impulsivity.

Other reasons for low correlations include differences in measurement approaches. Specifically, whilst temporal discounting involves choices made at a specific point in time with potentially real consequences at this moment (i.e., being informed of an immediate or later reward), self-report questionnaires typically ask about general cognitive, affective, and behavioral tendencies and preferences. Moreover, even when examining only temporal discounting, measurement methods differ. For example, time frames (e.g., date/delay effect; Read et al., 2005), reward magnitudes (e.g., magnitude effect), reward types (e.g., money, food, health etc.; Odum et al., 2020), number of trials (e.g., single-shot paradigms vs. comprehensive tasks involving several hundred trials), or measures derived from intertemporal choice tasks (e.g., discount rate vs. area under the curve) can lead to variation between temporal discounting tasks. The convergent validity of different measurement methods and factors has to be investigated further. It is likely, however, that such differences could contribute to inconsistent findings concerning the covariation with individual differences variables (i.e., differential external validity). A further issue that may lower the expected correlations is the task impurity problem (Miyake et al., 2000). This refers to the problem that any behavioral task measures not just the construct of interest, in this case sensitivity to magnitude and timepoint of reward, but also other, related or unrelated constructs. For example, intertemporal choice tasks may also tap into processing related to mathematical skills. The extent to which these account for correlations with other variables is unknown; latent factor modeling approaches may be instructive in this regard (Miyake et al., 2000).

Another important issue is related to risk. Rewards received in the future are always uncertain. However, temporal discounting tasks typically do not control for participants' perceived level of uncertainty of the rewards they are presented with, nor their level of risk aversion. Thus, studies of intertemporal choice ideally would simultaneously assess risk preferences and, potentially, preferences for early resolution of uncertainty to isolate intertemporal from risk preferences (Kreps and Porteus, 1978; Andersen et al., 2008; Lopez-Guzman et al., 2018, 2019).

Fourth, our review of the literature on cognitive styles showed that future studies would benefit from addressing the issue of task complexity. Additionally, a valuable approach would be to extend correlational designs in unselected samples to specific sub-populations with extreme expressions of relevant phenotypes, e.g., aphantasics or hyperphantasics (Zeman et al., 2020). Moreover, combinations of individual differences approaches with experimental challenges may be particularly informative in this context. Specifically, the interactive effects of cognitive styles such as future-orientation and theoretically related experimental manipulations such as episodic future thinking should be characterized.

Fifth, although numerous studies have shown steeper temporal discounting to be associated with lower IQ, multivariate designs are needed to reliably identify moderators of the relationships between IQ and temporal discounting. Such work would be important to clarify why the observed correlations emerge.

Sixth, functional neuroimaging has produced replicable findings of the neural activation patterns accompanying intertemporal choice at group-level (Peters and Büchel, 2011; Frost and McNaughton, 2017). However, there is less work on correlations between discounting and measures of brain function as well as structure, and findings are generally inconsistent. These inconsistencies likely arise in part from low power due to small sample sizes and the low reliability of some of the obtained measures. Larger samples may not, however, be the panacea in this field. A host of issues from task-related factors such as difficulty to sample-related factors such as levels of impulsivity have to be considered to aid interpretation of the direction and strength of correlation.

Seventh, studies have begun to shed light onto the molecular genetics of temporal discounting. Whilst initial candidate gene studies have been seminal in launching this work, they tended to suffer from low power to detect the likely very small effects of individual gene variants and they lack independent, large-scale replications. Indeed, later GWAS approaches have not confirmed these early findings. Generally, this work is most likely to progress through large-scale studies, such as multi-center consortia (Thompson et al., 2014). These large-scale studies are also needed to assess possible genetic links between temporal discounting and psychological variables.

Specific suggestions for future genetic studies also include the observation that temporal discounting is a complex phenotype. Therefore, future work may wish to focus instead on subprocesses or neural measures, e.g., activation levels in specific areas of the temporal discounting network. Additionally, it is likely that genetic effects depend on environmental variables; therefore, gene × environment interactions should be considered in future studies.

## Conclusions

The study of temporal discounting is a mature research field that has produced a number of replicable experimental (Rung and Madden, 2018) and neuroimaging (Frost and McNaughton, 2017) findings. Individual differences approaches have also generated some replicable findings; however, the field is still developing and many important questions are still unexplored.

General recommendations to the field include the issue of statistical power; correlations tend to be of small magnitude, necessitating large samples, clear a priori hypotheses, and independent replications. Given the task impurity problem, the field may also benefit from application of latent trait analyses; these have been applied with success in other areas of cognitive psychology (Friedman and Miyake, 2004). Fundamentally, evidence of reliability from test–retest studies should be supplemented by LST modeling, to properly separate trait from state variance and measurement error, as a fundamental basis for further individual differences work.

A final recommendation is to combine individual differences and experimental approaches. Various experimental manipulations reliably alter discount rates, including episodic future thinking manipulations or framing effects (Rung and Madden, 2018). Individual differences in these experimental effects, however, remain to be systematically explored. Such work would also be able to provide a more fine-grained picture of the patterns of correlations between temporal discounting under different conditions and personality, cognitive or neurobiological variables.

## Author Contributions

UE, CM, and BW developed the idea of the review. KK, QR, CM, and UE decided on the concrete conception and did the literature research. KK, QR, CM, and UE wrote the manuscript. All authors contributed to the article and approved the submitted version.

## Conflict of Interest

The authors declare that the research was conducted in the absence of any commercial or financial relationships that could be construed as a potential conflict of interest.

## Footnotes

^1^Since Parthasarathi et al. (2017) did not conduct repeated-measures ANOVA, only effect sizes of differences in discount rates between the groups before (*p* = 0.09, *d* = 0.17) and after (*p* = 0.04, *d* = 0.71) training can be reported.

^2^Candidate-gene studies have been reported to additionally suffer from other more general issues. Considering the scope of our review, we do not discuss these issues in detail but instead refer the reader to Tabor et al. (2002), Zhu and Zhao (2007), Hart et al. (2013), and Farrell et al. (2015).
